# Concomitant Use of Sulforaphane Enhances Antitumor Efficacy of Sunitinib in Renal Cell Carcinoma In Vitro

**DOI:** 10.3390/cancers14194643

**Published:** 2022-09-24

**Authors:** Igor Tsaur, Anita Thomas, Emine Taskiran, Jochen Rutz, Felix K.-H. Chun, Axel Haferkamp, Eva Juengel, Roman A. Blaheta

**Affiliations:** 1 Department of Urology, University Medical Center, Johannes Gutenberg University, 55131 Mainz, Germany; 2 Department of Urology, University Hospital Frankfurt, Goethe University, 60318 Frankfurt, Germany

**Keywords:** renal cell carcinoma, sulforaphane, sunitinib, growth, proliferation, complementary alternative medicine (CAM)

## Abstract

**Simple Summary:**

Despite recent advances in treating metastatic renal cell carcinoma (RCC), many patients develop resistance to therapy, resulting in treatment failure. Sunitinib is one drug used to treat metastasized RCC and resistance eventually develops in most patients. In the present in vitro investigation, sulforaphane, a natural compound known to possess antitumor properties without inducing severe side effects, enhanced the efficacy of sunitinib by preventing tumor growth and proliferation in sunitinib-resistant RCC. Sulforaphane, therefore, could prove beneficial as an integrative component in treating metastasized RCC with sunitinib. Further investigation is required to verify these in vitro findings and to evaluate sulforaphane’s clinical value.

**Abstract:**

Chronic treatment of renal cell carcinoma (RCC) with the tyrosine kinase inhibitor sunitinib (ST) inevitably induces resistance and tumor re-activation. This study investigated whether adding the natural compound sulforaphane (SFN) with its anti-cancer properties could improve ST efficacy in vitro. The RCC cell lines A498, Caki1, KTCTL26, and 786O were exposed to ST, SFN, or both (dual therapy, DT) before (short-term exposure) and during ST-resistance buildup (long-term 8-week exposure). Tumor growth, proliferation, and clone formation were evaluated, as was cell cycle progression and cell cycle regulating proteins. In nonresistant cells (short-term), DT induced a higher reduction in cell viability in three cell lines as compared to monotherapy with either ST or SFN. Long-term SFN or DT significantly reduced tumor growth and proliferation, whereas ST alone had no effect or even elevated proliferation in three cell lines. SFN or DT (but not ST alone) also blocked clonogenic growth. Both long-term SFN and DT enhanced the number of cells in the S- and/or G2/M-phase. Protein analysis in 786O cells revealed a down-regulation of cyclin dependent kinase (CDK) 1 and 2. CDK2 or Cyclin A knockdown caused reduced 786O growth activity. SFN therefore inhibits or delays resistance to chronic ST treatment.

## 1. Introduction

In the year 2020, renal malignancies accounted for 2.2% of new tumor cases and 1.8% of cancer deaths worldwide [1]. A recent analysis of the National Cancer Database has demonstrated an increase in the rate of metastatic renal cell carcinoma (RCC) by a third in only 7 years [2]. In particular, a growing trend in RCC-related mortality necessitates further optimization to prevent and treat this cancer [3].

Sunitinib (ST), a tyrosine kinase inhibitor (TKI), has been shown to significantly extend the progression-free survival (PFS) in previously untreated patients with metastatic (m)RCC, compared to those receiving interferon alfa [4]. After the introduction of ST in 2006, it, along with other targeting drugs, has gradually become the backbone of mRCC management. Due to the recent introduction of immune checkpoint inhibitors (ICIs), standard care for clear cell mRCC has shifted to combination regimens including ICI/ICI or ICI/TKI since these treatments are superior to that with ST alone [5]. Nevertheless, ST still remains important in managing mRCC patients not eligible for or not tolerating ICIs or for those with non-clear cell mRCC [5]. In addition, it has been approved by the Food and Drug Administration (FDA) for adjuvant treatment of high-risk mRCC following nephrectomy [6].

The PFS and response rate to ST for mRCC derived from real-world data are 11 months and 35% at most [7], respectively. Thus, improving the efficacy of ST monotherapy without an increase in toxicity has fostered the search for additive therapeutic partners. Due to their antineoplastic effects and favorable toxicological portfolio, phytochemicals are increasingly being brought into cancer therapy [8]. Sulforaphane (SFN) is a natural isothiocyanate modulating various cellular targets involved in tumorigenesis [9], and we aimed to assess its impact on the neoplastic behavior of RCC cells when added to ST.

It has recently been documented that TKIs not only influence endothelial angiogenesis but also directly affect cancer cells by altering intracellular signaling cascades involved in tumor growth regulation [10]. This is important, since TKI resistance and tumor progression have been associated with the activation of tyrosine kinases and downstream targets, promoting tumor cell proliferation and survival [11]. Since chemopreventive properties of SFN have been documented, the current study was based on the rationale that SFN might improve a failing TKI-based treatment regimen.

## 2. Materials and Methods

### 2.1. Cell Culture

RCC cell lines Caki1 and KTCTL-26 were derived from LGC Promochem (Wesel, Germany). A498 cells were purchased from Cell Lines Service (Heidelberg, Germany), and 786O were obtained from Prof. Wilhelm Krek (Institute of Cell Biology Zürich, Switzerland). The tumor cells were grown in RPMI 1640 medium supplemented with 10% fetal calf serum (FCS), 2% HEPES (2-(4-(2-Hydroxyethyl)-1-piperazine)-ethanesulfonic acid) buffer, 1% Glutamax (all Gibco/Invitrogen, Karlsruhe, Germany), and 1% penicillin/streptomycin (both Sigma-Aldrich, München, Germany) at 37 °C in a humidified 5% CO_2_ incubator. 

### 2.2. Drugs

ST was purchased from LC Laboratories, Woburn, MA, USA, and SFN was provided by Biomol, Hamburg, Germany. Based on earlier dose–response analyses, tumor cells were treated with 1 μM ST, 5 µM SFN, or both (dual therapy, DT) and then evaluated shortly after drug exposure (short-term incubation) or after an 8-week pre-incubation with ST, SFN, or DT (long-term incubation). This 8-week period has been shown to induce ST-resistance [12,13]. The length of the short-term incubation varied according to the time taken to carry out different analyses. 

### 2.3. Cell Growth

Cell growth was measured by using dye 3-(4,5-dimethylthiazol-2-yl)-2,5-diphenyltetrazolium bromide (MTT). PeCa cells (50 µL, 1 × 10^5^ cells/mL) were seeded into 96-well plates. After 24, 48, and 72 h, 10 µL of MTT (0.5 mg/mL) (Roche Diagnostics, Penzberg, Germany) was added to each well. After 4 h, cells were lysed using sodium dodecyl sulfate (10% SDS in 0.01 M HCl). The plates were then incubated overnight at 37 °C. The absorbance at 570 nm was determined with a multi-well ELISA reader (Tecan Infinite M200, Männedorf, Switzerland). After subtracting background absorbance and matching with a standard curve, the results were expressed as mean cell number. The mean cell number was set at 100% after 24 h of incubation.

### 2.4. Clonogenic Growth

Five hundred cells/well were seeded onto a 6-well-plate. Following 5–10 days of incubation without medium change, cell colonies were fixed and counted. Clones ≥50 cells were counted as one colony using a Zeiss ID 03 light microscope (Zeiss AG, Oberkochen, Germany).

### 2.5. Proliferation

Cell proliferation was measured using a BrdU (5-bromo-2′-deoxyuridine) cell proliferation enzyme-linked immunosorbent assay (ELISA) kit (Calbiochem/Merck Biosciences, Darmstadt, Germany). Tumor cells (50 μL, 1 × 10^5^ cells/mL) seeded onto 96-well microtiter plates were incubated with 20 μL of BrdU labeling solution per well for 24 h and subsequently fixed and detected using anti-BrdU mAb, according to the manufacturer’s instructions. Absorbance was measured at 450 nm using a microplate ELISA reader (Tecan Infinite M200, Männedorf, Switzerland). Values were expressed as a percentage compared to untreated controls, which were set to 100%.

### 2.6. Cell Cycle Analysis

Cell cycle analysis was carried out with subconfluent tumor cells. Tumor cell populations were stained with propidium iodide (Cycle TEST PLUS DNA Reagent Kit, BD Biosciences, Heidelberg, Germany) and then subjected to flow cytometry with a FACScan flow cytometer (BD Biosciences). Ten-thousand events were collected for each sample. Data acquisition was carried out using Cell-Quest software, and cell cycle distribution was calculated using the ModFit software (BD Biosciences). The number of gated cells in the G1, G2/M, or S-phase is presented as %.

### 2.7. Western Blot

Tumor cell lysates were applied to polyacrylamide gels and separated for 10 min at 80 V and for 60–90 min at 120 V. The proteins were then transferred to nitrocellulose membranes. After blocking with non-fat dry milk for 1 h, the membranes were incubated overnight with the following primary antibodies: CDK1 (mouse IgG1, clone 1, dilution 1:2500), pCDK1 (Mouse IgG2a, clone 55, dilution 1:2500), CDK2 (mouse IgG2a, clone 55, dilution 1:2500), Cyclin A (Mouse IgG1, clone 25, dilution 1:500), Cyclin B (Mouse IgG1, clone 18, dilution 1:1000), and p27 (Mouse IgG1, clone 57/Kip1, dilution 1:500) (all BD Biosciences) as well as pCDK2 (Rabbit, polyclonal antibody, dilution 1:1000) and p19 (Rabbit IgG, clone 12D1, dilution 1:1000) (both Cell Signaling). HRP-conjugated rabbit-anti-mouse IgG or goat-anti-rabbit IgG (IgG, both: dilution 1:1000, Dako, Glosturp, Denmark) served as secondary antibodies. The membranes were incubated with an ECL detection reagent (ECL; Amersham/GE Healthcare, München, Germany) and then analyzed by the Fusion FX7 system (Peqlab, Erlangen, Germany). β-Actin served as the internal control (1:1000; clone AC-15; Sigma-Aldrich, Taufenkirchen, Germany). The ratio of protein intensity/β-actin intensity was calculated for pixel density analysis of the protein bands and illustrated as percentage of controls (GIMP 2.8 software, www.gimp.org, accessed on 9 October 2021).

### 2.8. siRNA Blockade

Tumor cells (3 × 10^5^/6-well) were transfected with small interfering RNA (siRNA) directed against cyclin A (gene ID: 890, target sequence: GCCAGCTGTCAGGATAATAAA) and cdk2 (gene ID: 1017, target sequence: AGGTGGTGGCGCTTAAGAAAA) with a siRNA/transfection reagent (HiPerFect Transfection Reagent; Qiagen, Hilden, Germany) in 1:6 ratio. Cells treated with 5 nM control siRNA (All stars negative control siRNA; Qiagen, Hilden, Germany) were used as controls. Western blot analysis was used to confirm knock-down, and tumor cell growth was measured by the MTT assay as previously described.

### 2.9. Statistics

All experiments were performed ≥3 times. Statistical significance was determined with the Wilcoxon–Mann–Whitney U test or with ANOVA along with the Dunnett’s test. Differences were considered statistically significant at a *p*-value ≤ 0.05.

## 3. Results

### 3.1. Tumor Cell Growth and Proliferation

**Figure 1:**
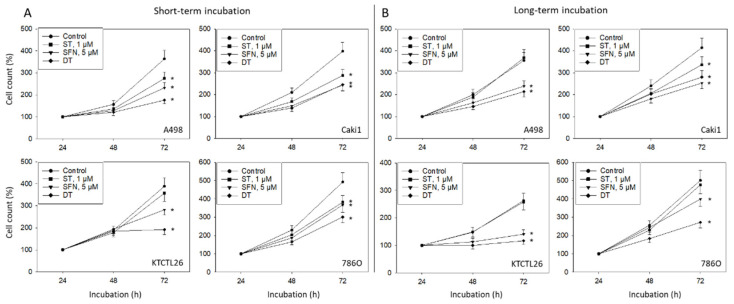
Influence of 1 µM sunitinib (ST), 5 µM sulforaphane (SFN), or both (DT) on cell growth in a panel of renal cell cancer cell lines. Cell lines were exposed to the compounds and evaluated after 24 (100%), 48, and 72 h by the MTT assay (short-term incubation, (**A**)), or they were preincubated with the compounds for 8 weeks and then re-exposed to them before being subjected to the MTT assay (long-term incubation, (**B**)). Cell number after 24 h is depicted in Supplement (Figure S1). Error bars indicate standard deviation, * indicates significant difference to untreated controls set to 100%. *n* = 6.

Short-term treatment with 1 μM ST induced a significant loss of A498, Caki1, and 786O (but not KTCTL26) cells, whereas short-term SFN application was associated with a reduced cell number in all cell lines, compared to their respective controls (Figure 1A). Interestingly, DT was superior to the single drug use in A498, KTCTL26, and 786O cells. ST lost its growth suppressive properties when exposed to A498, KTCTL26, and 786O cells for 8 weeks, whereas growth in all tumor cell lines was well suppressed after the 8 week long-term SFN application (Figure 1B). In 786O cells, a higher efficacy of the ST-SFN drug combination was observed, compared to SFN alone. 

**Figure 2:**
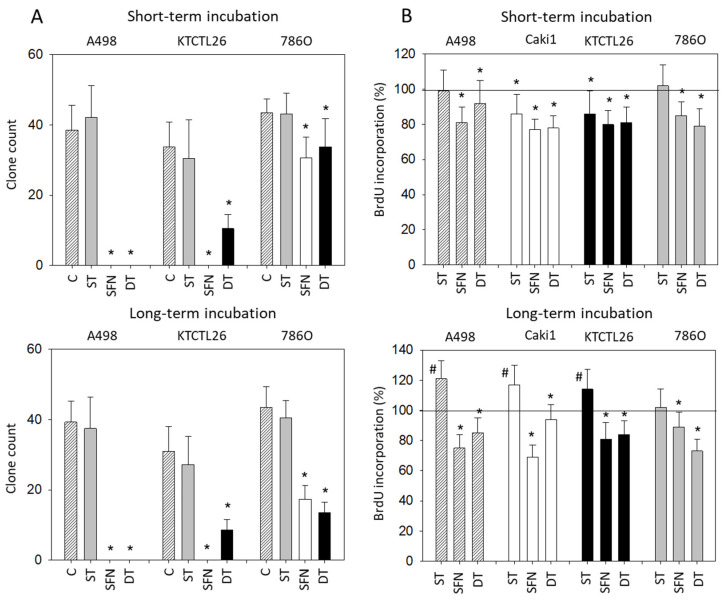
Cell clonogenic growth and proliferation. (**A**) Number of A498, KTCTL26, and 786O cell clones exposed to 1 µM sunitinib (ST), 5 µM sulforaphane (SFN), or both (DT). Cells were subjected to the assay shortly after drug exposure (short-term incubation) or after re-exposure following an 8 week pre-incubation (long-term incubation). Controls (C) are without drugs. Error bars indicate standard deviation, *n* = 3. * indicates significant difference to untreated controls. (**B**) Evaluation by BrdU incorporation. A498, Caki1, KTCTL26, and 786O cells were subjected to the assay immediately after drug application (short-term incubation) or following an 8 week pre-incubation (long-term incubation). Controls (C) are without drugs. Error bars indicate standard deviation. * Indicates significant down-regulation, # indicates significant up-regulation to untreated controls set to 100% (indicated by a black line). *n* = 3.

Clones were only evaluated on A498, KTCTL26, and 786O cell cultures, since Caki1 did not form clones. ST did not alter clone number, whether it was applied short- or long-term (Figure 2A). A marked clone loss was apparent in the presence of SFN or DT. 

ST only moderately reduced BrdU incorporation in Caki1 and KTCTL26 cells following short-term application (Figure 2B upper), whereas BrdU incorporation increased in all cell lines (excepting 786O) after chronic ST exposure for 8 weeks (Figure 2B lower). In contrast, both SFN and DT diminished BrdU uptake in all cell lines under short-term and long-term conditions. DT was even superior to the SFN mono-treatment in 786O cells when exposed chronically over 8 weeks (Figure 2B lower). 

### 3.2. Tumor Cell Cycling

**Figure 3:**
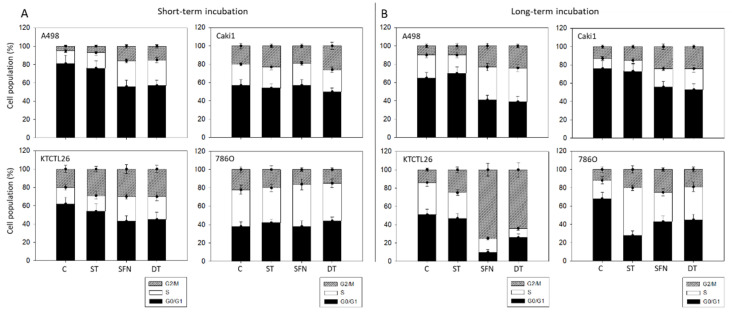
Influence of 1 µM sunitinib (ST), 5 µM sulforaphane (SFN), or both (DT) on proportionate G0/G1, S, and G2/M-phases of the cell cycle in A498, Caki1, KTCTL26, and 786O cells. (**A**) shows cells exposed short-term to the drugs, (**B**) shows cells pre-treated with the compounds for 8 weeks and subsequently re-exposed to the drugs (long-term incubation). Mean of *n* = 3 experiments.

Cell cycle analysis was inhomogenous. In the short-term, ST slightly elevated the amount of S- and G2/M-phase in A498 and KTCTL26 cells. This effect was stronger when SFN or DT were applied (Figure 3A). However, no distinct modification of Caki1 or 786O cell cycling was noted following single drug or DT use. Exposing the tumor cells chronically over 8 weeks to ST was associated with a moderate increase in G2/M-phase cells, paralleled by a decrease in S-phase in A498 and KTCTL26 cells. However, S-phase 786O cells considerably increased with ST, accompanied by a loss of G0/G1 phase cells (Figure 3B). Long-term effects of SFN and DT were similar in all cell lines with strong accumulation in the G2/M phase associated with a loss in the G0/G1 phase. 

### 3.3. Cell Cycle Regulating Proteins

**Figure 4:**
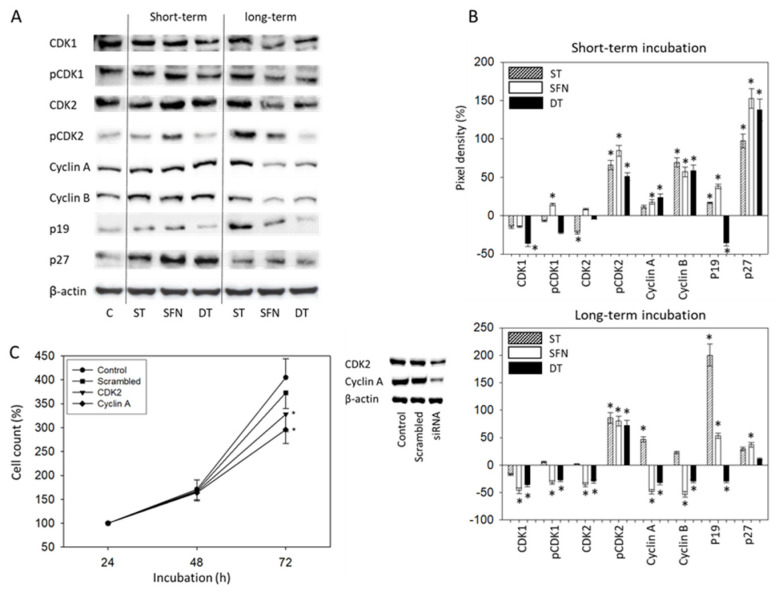
(**A**) Western blot of cell cycle related proteins from 786O lysates. Tumor cells were pretreated with 1 µM sunitinib (ST), 5 µM sulforaphane (SFN), or both (DT) for 24 h. Controls (**C**) remained untreated. β-actin served as the internal control. One representative from three separate experiments. (**B**) Ratio of protein intensity/β-actin intensity after short- (upper) or long-term (lower) drug incubation, expressed as a percentage of the controls, set to 0%. Error bars indicate standard deviation, *n* = 3. * indicates significant difference to controls. Original blots: see Supplementary Figure S1. (**C**) Cell growth of 786O cells treated with a CDK2 or Cyclin A specific siRNA, evaluated by the MTT-assay (versus untreated controls or scrambled siRNA). * Indicates significant difference to untreated controls set to 100%. (**C**) Right shows protein expression level following siRNA transfection (untreated control versus scrambled siRNA versus specific siRNA). Each protein analysis was accompanied by a β-actin loading control. One representative internal control is shown.

Final studies concentrated on the 786O cell line. Short-term ST application up-regulated p19 only moderately but p27 considerably, contrasting the long-term effects with a strong enhancement of p19 and no effects on p27 protein expression (Figure 4A,B). SFN short-term treatment was paralleled by strongly elevated pCDK2 and p27 and enhanced p19. SFN’s mode of action under long-term conditions differed in that CDK1 (both total and activated) and CDK2 along with their counterparts, Cyclin A and B, were potently diminished. However, pCDK2 remained high in cells chronically treated with SFN. A loss of CDK-cyclin proteins was also seen in the presence of chronic DT, except pCDK2, which was elevated, compared to the control. Since the CDK2-Cyclin A axis was strongly influenced by SFN and DT exposure, the relevance of both proteins was analyzed in siRNA knock-down studies (Figure 4C). Diminished protein expression caused by the specific siRNA (Figure 4C, right panel) correlated significantly with reduced 786O growth activity (Figure 4C, left panel).

## 4. Discussion

Besides immunotherapy, ST still plays a major role in managing mRCC, and strategies aimed at enhancing and prolonging ST efficacy are still sought. Treatment-related adverse events are encountered in up to 95% of mRCC patients treated with ST, preventing its combination with many other antitumor compounds whose administration is accompanied by adverse side effects as well [14]. Combining ST with natural products that have been shown to possess antitumor potential and, by nature good tolerability, might be an alternative. We hypothesized that concurrent application of ST with SFN might potentiate the anticancer activity of the agents alone. The results presented here show that the effect of SFN and DT on ST-sensitive cells (short-term incubation) was superior to that of ST by reducing cell growth and proliferation.

After ST had lost its antitumor effect as a result of resistance development following long-term ST-exposure, both DT and SFN still maintained their antitumor properties. Tumor clone formation could still be dramatically reduced through DT and SFN treatment. In ST-resistant A498, Caki1, and KTCTL26 cells proliferative activity was even elevated after renewed ST application, which may be an indication of drug resistance. Applying SFN and DT still resulted in down-regulation of proliferative activity. In ST-resistant 786O cells DT was superior to SFN alone in suppressing cell growth. This was reflected in cell cycle alteration and suppressed clone formation and may indicate re-sensitization towards ST in this cell line. Other investigators have also reported that SFN reverses chemo-resistance to temozolomid, to cisplatin, and to the TKI lapatinib [15,16,17]. Accordingly, these observations indicate that combining ST with SFN could delay or revert resistance to ST. 

The study protocol was based on an ST concentration of 1 µM. Gotink et al. has developed resistant 786O cells by continuously exposing them to increasing ST concentrations, up to 6 µM [18]. Similarly, Adelaiye et al. has produced ST resistant 786O cells by treating the cells with an increasing ST concentration, up to 5 µM [19]. We observed rapid cell loss when the tumor cells were exposed long-term to an ST dosage ≥ 2 µM. Therefore, 1 µM ST, which is considered to be clinically relevant [18], served as the working concentration in our model. 

In the present investigation, an additive effect of DT on A498, Caki1, and KTCTL26 cell growth in the short-term treatment model was observed, as compared to either monotherapy. However, this additive inhibitory effect did not hold true for clonogenic growth since DT was not superior to SFN in preventing clonogenic cell proliferation. Interestingly, strong loss of the clone number of A498 and KTCTL26 cells in the presence of SFN or DT was observed, whereas only a moderate influence was evoked in 786O cells (Caki1 cells did not form clones). A similar response pattern became obvious with the cell cycle analysis, whereby a distinct accumulation of A498 and KTCTL26 cells in the S- and G2/M-phase in the presence of SFN or DT was apparent, whereas only minor effects were induced in 786O cells. It is concluded that the drugs’ influence on cell cycling may at least in part be responsible for blocking tumor progression in terms of clonogenic growth. Remarkably, SFN and DT influenced tumor cell cycling in a similar manner, indicating that the effect of SFN predominates over any effect of ST.

Under long-term application, DT was superior to SFN in reducing the clone count and growth of 786O cells; however, the differences were not statistically significant. Considering that both DT and SFN acted equally well on A498, Caki1, and KTCTL26 cells, it appears that both SFN and DT maintain their oncosuppressive properties in drug-resistant cells in contrast to ST alone. Possibly, ST activates undesired feedback mechanisms, leading to resistance development. This could provide a DT regimen with a considerable advantage over ST alone.

In accordance, a significant attenuation of viability of gastric cancer cells has been reported when treated with SFN combined with the TKI lapatinib, compared to either monotherapy [20]. Importantly, the effect of SFN on cell viability was not diminished in the lapatinib-resistant cells, corroborating the rationale of combining TKIs with SFN. The combined use of SFN and lapatinib was associated with the accumulation of both treatment-naïve and -resistant cells in the G0/G1-phase (18), contrasting with our results, which reveal an elevation in the cells in the S- and G2/M-phase in the presence of SFN and DT. Our data are in accordance with others pointing to a G2/M arrest as a principal mechanism of SFN [21,22]. However, the response of tumor cells to combined SFN-TKI treatment may also depend on the particular TKI and the tumor cells used. 

The increase in 786O cells in the S- and G2/M-phase upon long-term exposure to DT or SFN was accompanied by a loss of total CDK2 along with its complex partner Cyclin A. The same was seen with CDK1 (both total and phosphorylated) and Cyclin B. This observation is intriguing, since CDK2 is thought to control the intra S-phase checkpoint and to inhibit cell cycle exit induced by DNA damage reduction [23]. Thus, a reduction in CDK2 may delay S/G2 progression after DNA damage and mediate an exit from the cell cycle in G2. Since DNA damage has been reported to be provoked by SFN in osteosarcoma cells [24], down-regulation of CDK might explain how DT and SFN promote a cell cycle exit, diminishing cell growth and proliferation. 

The reduction in CDK1 and Cyclin B is clinically relevant. CDK1 has been found to be closely associated with RCC prognosis [19]. Moreover, CDK1 has been defined as a powerful predictor of RCC recurrence and as a regulator of RCC proliferation, migration, and invasion [20]. Down-regulation of CDKs and the corresponding Cyclins might, therefore, be responsible for the suppression of RCC cell growth and proliferation seen under long-term treatment with either SFN or DT. 

Surprisingly, we observed an up-regulation of phosphorylated CDK2 after chronic treatment with each drug alone or with DT. It is not clear whether this is due to an undesired feedback mechanism or to an unspecific epiphenomenon. ST has been reported to be capable of directly binding to CDK2 [25,26] with subsequent CDK down-regulation and G1 arrest in acute myeloid leukaemia cells [27]. Hence, CDK2 elevation might be indicative of a growing adaptation of cells to SFN and/or ST. In fact, CDK2 has been identified as a key driver of melanoma resistance against BRAF and Hsp90 inhibitors [28]. Remarkably, advanced cervical cancer with a higher expression of CDK2 as well as Cyclin A was linked to inferior survival, whereas both markers were down-regulated in response to chemotherapy [29]. 

Comparative analysis of SFN effects on ST-resistant A498, Caki1, and KTCTL26 cells revealed down-regulation of both CDK2 and pCDK2 in all cell lines [26], opening the question of whether a CDK2 increase in 786O cells might be a cell line specific phenomenon. Indeed, the response of 786O cells to chronic SFN exposure in terms of cell growth reduction was minor, compared to the response of A498, Caki1, and KTCTL26 cells (Figure 1). In addition, 786O cells have been shown to express HIF-2α, HIF-1β, mechanistic Target of Rapamycin (mTOR), and BRCA1 genes as well as pmTOR proteins to a higher extent than A498 and Caki1 cells [27]. These investigators concluded that the differences in gene and protein expression may at least partially be responsible for different responses to several anticancer drugs added to the cell cultures [27]. Whether a similar scenario may hold true with respect to SFN and DT is not clear. It should be considered that CDK1-Cyclin B were strongly suppressed in 786O cells by SFN and DT, which may compensate for pCDK2 elevation. With particular respect to SFN, continuous exposure of pancreatic cancer cells to SFN did not induce drug resistance [30], making it likely that at least elevation of pCDK2 by SFN may not be associated with resistance. This, however, is speculative and requires further evaluation. 

Cyclin A was elevated in response to chronic therapy with ST, whereas the opposite was observed in the presence of both SFN and DT. Up-regulation of Cyclin A has already been observed in ST-resistant Caki1 cells [31]. The relevance of the CDK2-Cyclin A axis was verified by the siRNA knock-down analysis, revealing the close association between expression level and growth activity. An augmented expression of the Cyclin A1 gene has been associated with cellular resistance to paclitaxel, doxorubicin, and 5-fluorouracil in ovarian cancer [32]. In prostate cancer, the expression of Cyclin A1 has been shown to be enhanced in metastatic lesions compared to the primary tumor, while its over-expression in stem-like cells fostered bone marrow homing and metastatic growth [16]. In addition, augmented expression of Cyclin A in epithelial ovarian cancer has been associated with unfavorable tumor characteristics and a higher resistance to platinum-based chemotherapy [33]. We, therefore, assume that DT and SFN exert their antineoplastic activity, at least in part, by suppressing the CDK2/Cyclin A and CDK1/Cyclin B complexes—all major drivers of tumorigenesis and progression.

## 5. Conclusions

Evidence is provided in vitro that combining ST with SFN could increase the efficacy of ST in treating advanced RCC by inhibiting the resistance encountered with chronic ST application. The inhibition might be due to down-regulation of the CDK2/Cyclin A complex. These in vitro findings require verification in vivo. Additional experiments should also explore combining SFN with other TKIs as well as with ICIs.

## Data Availability

All data generated or analyzed during this study are included in this published article and its Supplementary Materials.

## Supplementary Materials

The following supporting information can be downloaded at https://www.mdpi.com/article/10.3390/cancers14194643/s1, Figure S1: Original western blots.
