# Supplementary figures and images for: Concomitant Use of Sulforaphane Enhances Antitumor Efficacy of Sunitinib in Renal Cell Carcinoma In Vitro
Source: Cancers (Basel). 2022 Sep 24;14(19):4643. doi: 10.3390/cancers14194643 (PMC9562895; doi:10.3390/cancers14194643)

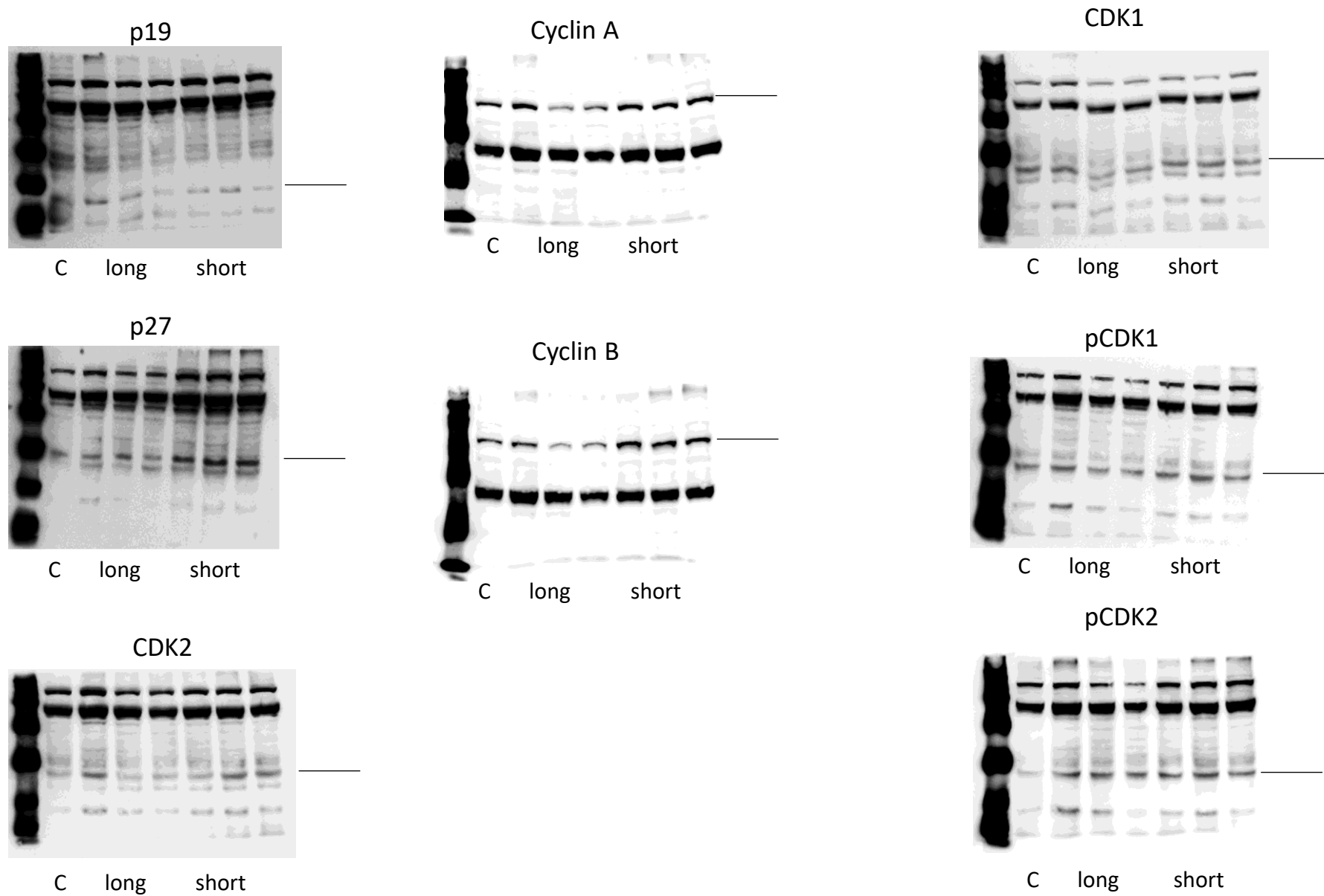

Figure S1: Original western blots.

Supplement: Supplementary file 1 [file cancers-14-04643-s001.zip › cancers-1891148-supplementary.pdf]
